# Activation of dopamine D4 receptors in the nucleus accumbens shell increases motivation for palatable food and homeostatic feeding, whereas systemic blockade reduces feeding behavior in rats

**DOI:** 10.3389/fpsyg.2026.1788044

**Published:** 2026-08-31

**Authors:** Juan Gabriel Tejas-Juárez, Josué Omar Suárez-Ortiz, Rodrigo Erick Escartín-Pérez, Juan Manuel Mancilla-Diaz, Josué Vidal Espinosa-Juárez, Alfredo Briones-Aranda, Fanny Rodríguez-Cruz, Eduardo Martínez-Abundis, Nancy Patricia Gómez-Crisóstomo, Refugio Cruz-Trujillo

**Affiliations:** 1 División Académica Multidisciplinaria de Comalcalco, Universidad Juárez Autónoma de Tabasco (UJAT), Comalcalco, Mexico; 2 Laboratorio de Neurobiología de la Alimentación, Grupo de Investigación en Nutrición, División de Investigación y Posgrado, FES Iztacala, UNAM, Tlalnepantla, EDOMEX, Mexico; 3 Escuela de Ciencias Químicas, Universidad Autónoma de Chiapas (UNACH), Ocozocoautla de Espinosa, Mexico; 4 Pharmacology Laboratory, Faculty of Human Medicine, Autonomous University of Chiapas, Tuxtla Gutiérrez, Mexico; 5 Departamento de Biología Celular, Centro de Investigación y de Estudios Avanzados del Instituto Politécnico Nacional (CINVESTAV), Ciudad de México, Mexico; 6 Departamento de Químicos Farmacobiólogos, Grupo de Investigación en Salud y Metabolitos Especializados con Potencial Farmacológico, Universidad Pablo Guardado Chávez (UPGCH), Tuxtla Gutiérrez, Mexico

**Keywords:** appetite regulation, conditioning operant, L-745, 870, PD-168, 077, stereotaxic techniques

## Abstract

**Objective:**

The nucleus accumbens shell is a key region involved in motivation and reinforcement behavior associated with the consumption of palatable foods. However, it has not been studied whether D4 dopaminergic receptors regulate homeostatic feeding and reward-dependent behaviors. The objective of this study was to evaluate the effects of activation of D4 dopaminergic receptors within nucleus accumbens shell and systemic D4 blockade on motivation for palatable food and homeostatic feeding in male Wistar rats.

**Methods:**

Adult male Wistar rats were tested in operant conditioning paradigms using sucrose pellets under fixed-ratio (FR1 and FR5) and progressive-ratio schedules to assess motivation for palatable food. Homeostatic feeding was evaluated by measuring standard chow intake in satiated rats during the first hour of the dark phase. Using stereotaxic surgery, a unilateral guide cannula was implanted into the NAcSh for the microinjection of the selective D4R agonist *PD-168,077* (0.1 μg) and the D4R antagonist L-745,870 (0.1 μg). Additionally, PD-168,077 (1 mg/kg, i.p.) and L-745,870 (0.1 mg/kg, i.p.) were administered systemically. Lever presses, reinforcers earned, breakpoint, and chow intake were recorded.

**Results:**

Intra-nucleus accumbens shell injection of PD-168,077 significantly increased motivation for palatable food, as reflected by greater lever pressing, reinforcers earned, and breakpoint, and increased standard chow intake. These effects were abolished by local pretreatment with L-745,870. Systemic administration of L-745,870 reduced both chow intake and operant responding for palatable food, decreasing lever pressing and breakpoint.

**Conclusion:**

Dopamine D4 receptors in the nucleus accumbens shell promote both motivational and consummatory aspects of feeding. In contrast, systemic D4 receptor blockade suppresses homeostatic feeding and motivation for palatable food, supporting an important role for D4 receptors in the regulation of feeding behavior.

## 1 Introduction

The sustained increase in the prevalence of overweight and obesity represents a major global public health problem and is closely associated with chronic degenerative diseases that increase morbidity and mortality (World Health Organization, 2024). Food intake is regulated by at least two complex and interacting brain circuits: the homeostatic circuitry, which is essential for maintaining body weight and metabolic balance, and hedonic circuitry, which is driven by the sensory properties and pleasurable value of food (reward). These systems coexist and are often simultaneously engaged during food consumption (Pais et al., 2025; Rossi and Stuber, 2018).

The interaction between food intake, reward processing, and compulsive feeding behavior has been primarily associated with the mesolimbic dopaminergic pathway, which originates in the ventral tegmental area (VTA) and projects to the nucleus accumbens (NAc). The NAc is anatomically and functionally subdivided into the core and the shell. The nucleus accumbens shell (NAcSh) maintains reciprocal connections with subcortical limbic regions, including the lateral hypothalamus (LH) and the VTA, enabling the integration of motivational, emotional, and metabolic signals involved in the regulation of feeding behavior (Heimer et al., 1997). Notably, the NAcSh is embedded within both hedonic and homeostatic feeding circuits, integrating information from these systems to modulate behavioral output (Marinescu and Labouesse, 2024).

The NAcSh receives homeostatic signals from hypothalamic nuclei, particularly from the LH, establishing an integrative circuit that links energy status with motivational drive toward food (Hsu et al., 2018). As a key component of neural circuits involved in reward processing and goal-directed behavior, the NAcSh functions as a central hub that integrates reward-related information through specialized neuronal ensembles and modulates decision-making processes (Prado et al., 2016; Arroyo et al., 2024). It receives reward-related inputs from the ventral hippocampus (vH), paraventricular nucleus of the thalamus, basolateral amygdala (BLA), VTA, and ventral pallidum, as well as signals related to food palatability and liking from the hippocampus and the opioid system (Marinescu and Labouesse, 2024). According to the incentive salience theory, food reward is not a unitary process but comprises at least two dissociable psychological components: hedonic impact (“liking”) and incentive salience (“wanting”). “Liking” refers to the affective and pleasurable properties of a reward, whereas “wanting” refers to the motivational process by which mesocorticolimbic circuitry attributes incentive salience to rewards and reward-associated cues, promoting food seeking and consumption, either consciously or unconsciously. Incentive salience involves the transformation of reward-related perceptions and representations by imbuing them with motivational salience, making stimuli more attractive and desirable, and thereby increasing their capacity to elicit approach and reward-seeking behavior (Robinson and Berridge, 1993; Morales and Berridge, 2020; Guillaumin et al., 2023).

Consumption of highly palatable or energy-dense foods induces dopamine release in the NAcSh, thereby enhancing pleasure and reinforcement (Morgane et al., 2005; Leung and Lutfy, 2025). Complementarily, a voltammetry study demonstrated that predictive cues signaling sucrose availability are sufficient to evoke dopamine release in this structure, even in the absence of immediate consumption (Roitman et al., 2008).

The NAcSh expresses dopamine receptors of the D1-like (D1 and D5) and D2-like (D2, D3, and D4) families (Lu et al., 1998; Sibley et al., 1993). In this context, medium spiny neurons (MSNs) expressing dopamine D1 receptors project to GABAergic neurons in the LH, promoting both appetitive (wanting) and consummatory aspects of feeding behavior (O'Connor et al., 2015; Jennings et al., 2014). While D1 and D2 receptors have been extensively studied in the regulation of both homeostatic and hedonic feeding (Guillaumin et al., 2023; Leung and Lutfy, 2025), the dopamine D4 receptor (D4R) exhibits preferential localization in the NAcSh (Svingos et al., 2000) and plays a key modulatory role in cortico-striatal glutamatergic neurotransmission (Ferré et al., 2022). However, its specific contribution to feeding regulation within the homeostatic component, remains poorly understood.

Dysfunction of D4Rs has been associated with several neuropsychiatric disorders, including attention-deficit/hyperactivity disorder, schizophrenia, psychostimulant addiction, mood disorders, eating disorders, and obesity (Botticelli et al., 2020). In humans, genetic evidence also supports a role for D4Rs in feeding-related behaviors. Polymorphisms of the DRD4 gene, particularly the 7-repeat allele (DRD4-7R), have been associated with a greater preference for and intake of calorie-dense foods compared with individuals lacking this variant (Paquet et al., 2021). This polymorphism has been linked to altered intracellular response to dopamine, which can lead to increased dopamine levels through excessive drug use or consumption of palatable foods (Botticelli et al., 2020). Moreover, the DRD4-7R allele has been associated with obesity in adulthood, which may originate from food choices made during the preschool years (Silveira et al., 2014). Although these studies evaluate naturally occurring genetic variation rather than pharmacological manipulation of D4Rs, they provide convergent evidence that alterations in D4R function are associated with reward-related eating behaviors and support the translational relevance of investing D4R signaling using selective pharmacological ligands in preclinical models.

In this context, the administration of selective D4R agonists and antagonists are a valuable pharmacological tool for elucidating the functional involvement of these receptors in modulating MSN activity within the NAcSh. Preclinical studies have demonstrated that the intra-NAcSh administration of the selective D4R agonist PD-168,077 increases the intake of highly palatable food intake in pre-satiated rats under a free-access paradigm (Cruz-Trujillo et al., 2024). Conversely, intra-NAcSh administration of the selective D4R antagonist L-745,870 significantly reduces active lever presses, the number of reinforcers earned, and the breakpoint under a progressive ratio schedule, indicating a reduction in the motivational drive to obtain palatable food (Cruz-Trujillo et al., 2020). However, these studies were restricted to local pharmacological manipulations within the NAcSh, leaving unresolved whether systemic administration of selective D4R ligands produces comparable effects on feeding behavior. Addressing this question is particularly relevant because systemic administration more closely resembles the route by which pharmacological interventions would be delivered in clinical settings and therefore provides greater translational relevance. Furthermore, the specific contribution of D4Rs to the homeostatic and motivational components of feeding behavior remains poorly understood. To address this question, the present study combined two complementary experimental paradigms. Operant training under a progressive ratio schedule was used to assess the motivational component of feeding (“wanting”) by measuring the effort rats were willing to exert to obtain sucrose pellets, an established index of reinforced efficacy (LeSage et al., 2006; Khan et al., 2025; Stamos et al., 2023). In parallel, food intake measured in the home cage during the dark phase following both intra-NAcSh and systemic administration of D4R ligands was used to evaluate the homeostatic component of feeding under physiological conditions (Tejas-Juárez et al., 2014; Maxwell et al., 2024).

The present study aimed to evaluate the physiological role of NAcSh D4Rs in the regulation of feeding by examining both homeostatic food intake and motivational responding for sucrose pellets in satiated male Wistar rats. We hypothesize that D4Rs activation in the NAcSh differentially modulates these two components of feeding behavior. Our results may contribute to the identification of novel therapeutic targets for managing obesity and eating disorders.

## 2 Material and methods

### 2.1 Animals

Adult male Wistar rats (220–240 g) were obtained from the in-house breeding colony maintained at the Vivarium of the Facultad de Estudios Superiores Iztacala (FES-Iztacala), Universidad Nacional Autónoma de México (UNAM). Only male rats were used to maintain consistency with our previous studies investigating the role of D4Rs in feeding behavior (Cruz-Trujillo et al., 2020, 2024) and to reduce potential variability associated with hormonal fluctuations. Rats were group-housed (five animals per cage) in acrylic home-cages under a 12-h inverted light/dark cycle at a controlled temperature (22 ± 2 °C), with *ad libitum* access to food and water unless otherwise specified. All experimental procedures were conducted during the dark phase. Experimental protocols were approved by the Ethical Committee of FES-Iztacala UNAM (CE/FESI/112017/1221) and were performed in accordance with the ethical guidelines in the Technical Specifications for the Production, Care, and Use of Laboratory Animals as established in the Official Mexican Norm for Animal Care (NOM-062-ZOO-1999).

### 2.2 Behavioral assessment of food-reinforced and homeostatic feeding

**Figure 1:**
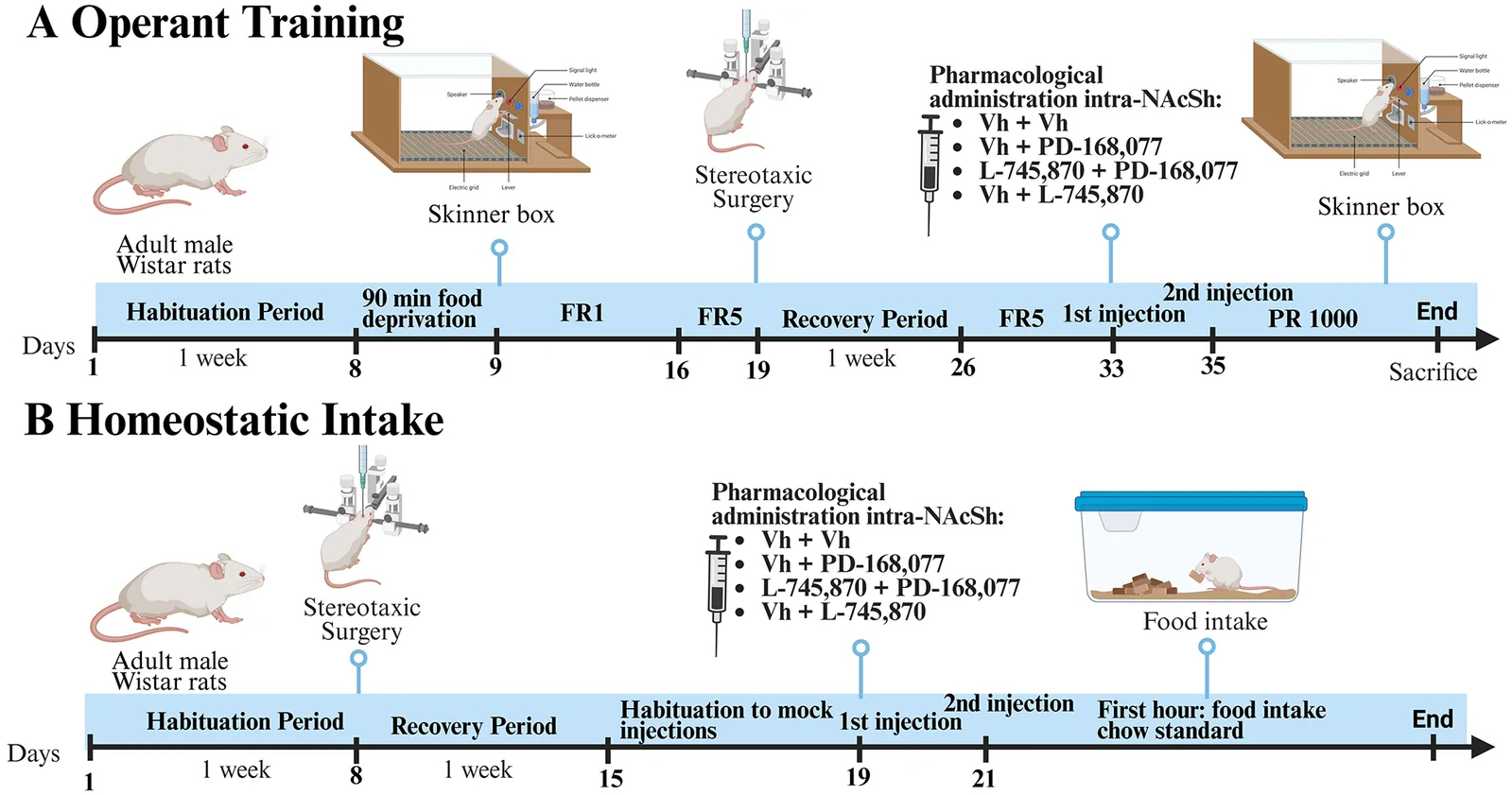
Experimental design for assessing operant and homeostatic food intake. **(A)** Operant training: Adult male Wistar rats were first habituated, followed by a 90-min period of food deprivation; immediately thereafter, they were placed in Skinner boxes and trained under FR1 and FR5 schedules. The animals then underwent stereotaxic surgery to implant cannulas targeting the NAcSh, followed by a 1-week recovery period. After the recovery period, training under the FR5 schedule resumed, and intra-NAcSh pharmacological administration was performed. Finally, food motivation was assessed using PR1000. **(B)** Homeostatic intake: A separate group of adult male Wistar rats were habituated and subjected to stereotaxic surgery for cannula implantation into the NAcSh. Then, the rats underwent a recovery period and habituation to mock injections. Then, the animals received intra-NAcSh pharmacological administrations, and their standard chow intake was measured during the first hour following injection. The timelines indicate the duration of each experimental phase in days. Created in https://BioRender.com.

Following 5 days of habituation to the housing room and daily handling by the experimenter, the rats were individually housed in acrylic home-cages (27 x 37 x 15 cm). The animals were fed standard laboratory chow (26.6% protein, 16.5% fat, and 56.8% carbohydrates, LabDiet^®^, Lab supply, Richmond, IN, USA). Rats were not maintained under chronic food restrictions. Instead, to enhance motivation for sucrose pellets reinforcement, animals underwent a 90-min period of acute food deprivation at the onset of the dark phase before each operant training and testing session from day 9 through day 19. Following each session, rats were returned to their home cages with *ad libitum* access to standard chow and water. Behavioral testing was conducted in operant chambers (Med Associates, Georgia, VT, USA). For homeostatic feeding experiments, rats were not food deprived. Standard chow was removed only during drug administration and was returned immediately afterward, allowing food intake to be measured over the subsequent dark phase (Figure 1), as described below.

#### 2.2.1 Operant behavior with intra-nucleus accumbens shell surgery

Rats were trained in daily 30-min sessions under a fixed ratio 1 (FR1) schedule, in which each press on the active lever resulted in the delivery of one sucrose pellet (reinforcer). Animals typically require approximately 8–10 training sessions to achieve stable responding. Stable responding was defined as ≥ 80% of total responses occurring on the active lever during three consecutive sessions. Once this criterion was achieved, rats were shifted to a fixed ratio 5 (FR5) schedule for three consecutive days. Following FR5 training, a subset of animals underwent stereotaxic surgery. Rats were anesthetized with a ketamine/xylazine mixture (Laboratorios Aranda/PiSA Agropecuaria, Mexico; 112.5/22.5 mg/kg, i.p.) and placed in a stereotaxic apparatus. A unilaterally stainless-steel guide cannula (23 G, 15 mm long; BD Precision Glide, Becton Dickinson and Co., Franklin Lakes, NJ, USA) was implanted into the left hemisphere targeting the NAcSh (AP +1.5 mm, ML −0.6 mm from bregma, and DV −6.0 mm relative to the dura mater). A unilateral approach was selected to minimize damage while allowing pharmacological manipulation of the NAcSh. Previous studies from our laboratory using unilateral administration into the NAcSh have demonstrated reliable effects on feeding and motivational behavior (Cruz-Trujillo et al., 2020, 2024). The guide cannula was secured to the skull with two stainless-steel anchor screws and dental acrylic to ensure stable fixation throughout the experimental procedures. Immediately after surgery, rats received a subcutaneous injection of enrofloxacin^®^ (2.5 mg/kg) to prevent postoperative bacterial infections.

Animals were allowed to recover for 1 week before behavioral testing resumed. After recovery, the same 90-min food deprivation protocol was maintained during the post-surgical FR5 retraining sessions. Rats were retrained under the FR5 schedule until stable responding of at least 80% of total responses occurring on the active lever during three consecutive sessions. For graphical representation of baseline operant performance, only the last four stable RF1 or RF5 sessions were included. Animals were then transferred to a progressive ratio (PR) schedule for 30 min to assess motivational feeding behavior (Figure 1A). Under the PR schedule, the response requirement increased according to the following sequence: 1, 3, 5, 12, 18, 27, 40, 60, 90, 135, 200, 300, 450, 675, and 1,000 lever presses. During PR sessions, the breakpoint, total number of reinforcers obtained, and number of active lever presses were recorded as motivational behavior (Figure 1A).

#### 2.2.2 Operant behavior for the systemic pharmacological evaluation of L-745,870

To assess the systemic effects of D4R blockade on operant behavior, an additional group of rats followed the same behavioral training protocol described above, except that they did not undergo stereotaxic surgery. After reaching the acquisition criterion (≥ 80% stability in lever-press responses across three consecutive sessions under the FR1 schedule), animals were transitioned to the FR5 schedule. Once they again achieved ≥ 80% response stability for three consecutive sessions, they proceeded directly to PR testing following systemic drug administration. Since these animals did not undergo stereotaxic procedures, no postsurgical recovery period or retraining phase was required.

#### 2.2.3 Homeostatic feeding behavior with intra-nucleus accumbens shell surgery

After a 1-week habituation period to the housing room and daily handling by the experimenter, a separate group of rats underwent stereotaxic surgery for unilateral implantation of guide cannulas targeting the NAcSh, as described above. Following a 1-week postoperative recovery period, rats were individually housed in acrylic cages.

Baseline standard chow intake was recorded for five consecutive days under mock intracannular injection conditions to habituate the animals to handling and the injection procedures, thereby minimizing stress during the experimental sessions. Beginning on day six, pharmacological treatments were administered 20 min before the onset of the dark phase. Immediately after drug administration, a pre-weighed amount of standard chow (Lab Diet^®^) was placed in the food hopper of each cage. After 60 min, the remaining chow and any food spillage were collected and weighed to calculate standard chow intake. Homeostatic feeding behavior was quantified as the amount of standard chow consumed during the first hour of the dark phase of the inverted light/dark cycle (Figure 1B).

#### 2.2.4 Homeostatic feeding behavior for the systemic pharmacological evaluation of L-745,870 and PD-168,077

To assess the systemic effects of D4R activation and blockade on homeostatic feeding, an additional group of rats followed the same experimental protocol described above, except that they did not undergo stereotaxic surgery. Rats were habituated to handling for four consecutive days. Treatments were then administered according to a Latin square design under the following experimental conditions: vehicle, PD-168,077 (1 mg/kg) and L-745,870 (0.1 mg/kg). Each animal received all three treatments at different time points, with a washout period of at least 2 days between administrations to prevent carryover effects.

### 2.3 Experimental design and procedure

For intra-NAcSh pharmacological evaluations, rats were randomly assigned to four experimental groups following a Latin square design: (1) Vehicle + Vehicle; (2) Vehicle + *PD-168,077* (0.1 μg); (3) *L-745,870* (0.1 μg) + *PD-168,077* (0.1 μg); and (4) Vehicle + *L-745,870* (0.1 μg).

L-745,870 and PD-168,077 were obtained from Tocris Bioscience (Atlantic Road, Bristol, UK): PD-168,077 (Code: 1065; *N-[[4-(2-cyanophenyl)-1-piperazinyl]methyl]-3-methylbenzamide maleate salt*), L-745,870 (Code: 1002; *3-(4-[4-chlorophenyl]piperazin-1-yl)-methyl-1H-pyrrolo [2,3-b]pyridine trihydrochloride*). Stock solutions were prepared in 2.5% dimethyl sulfoxide (DMSO) and 97.5% sterile 0.9% saline. Drugs were subsequently diluted to the desired concentration, resulting in a final DMSO concentration of 0.5% in all injected solutions. The vehicle solution consisted of 0.9% saline containing the same final concentration of DMSO (0.5%), ensuring identical injection conditions across all experimental groups.

Intracannular doses were selected based on previous studies (Cruz-Trujillo et al., 2024). Each intracannular administration consisted of a total concentration of 0.1 μg of drug delivered in a final infusion volume of 0.3 μL. Rats received unilateral intracannular injections at an infusion rate of 0.1 μL/min using a high-precision Hamilton syringe (Hamilton Company, Reno, NV) connected to a 16-mm microinjector targeting the NAcSh.

Table 1 summarizes the experimental groups used for the dual intracannular administration protocol. During the first intracannular administration, animals received either vehicle (Groups 1, 2, and 4) or L-745,870 (Group 3). 10 min later, a second intracannular administration was performed with vehicle (Group 1), PD-168,077 (Groups 2 and 3), or L-745,870 (Group 4). Following each infusion, the injector remained in place for 1 min to allow drug diffusion and minimize reflux.

**Table 1:** Experimental groups under the Latin square design.

Group	First intracannular administration	Second intracannular administration	Pharmacological condition
1	Vehicle (0.9% saline + 0.5% DMSO)	Vehicle (0.9% saline + 0.5% DMSO)	Control
2	Vehicle (0.9% saline + 0.5% DMSO)	PD-168,077 (0.1 μg)	D4 receptor activation (agonist)
3	L-745,870 (0.1 μg)	PD-168,077 (0.1 μg)	D4 receptor blockade + activation (antagonist + agonist)
4	Vehicle (0.9% saline + 0.5% DMSO)	L-745,870 (0.1 μg)	D4 receptor blockade (antagonist)

The sequential administration allowed pretreatment with the antagonist before agonist administration to determine whether D4R blockade prevented the behavioral effects induced by receptor activation. Vehicle administrations were included to maintain identical handling conditions across all experimental groups. In all cases, intracannular injections were completed 20 min before the initiation of PR testing (food-reinforcement behavior) or standard chow exposure (homeostatic feeding). Immediately after the second injection, animals were placed in the operating chambers or feeding cages.

For systemic pharmacological evaluations of operant behavior, rats were randomly assigned to two independent groups: (1) vehicle and (2) L-745,870 (0.1 mg/kg, i.p.). The systemic dose of L-745,870 was selected based on Patel et al. (1997), who demonstrated that 0.1 mg/kg produces > 90% occupancy of brain D4R receptors. In the present study, L-745,870 was administered 20 min before PR testing or standard chow exposure to allow sufficient time for systemic drug distribution and receptor engagement prior to behavioral assessment.

### 2.4 Histological verification

**Figure 2:**
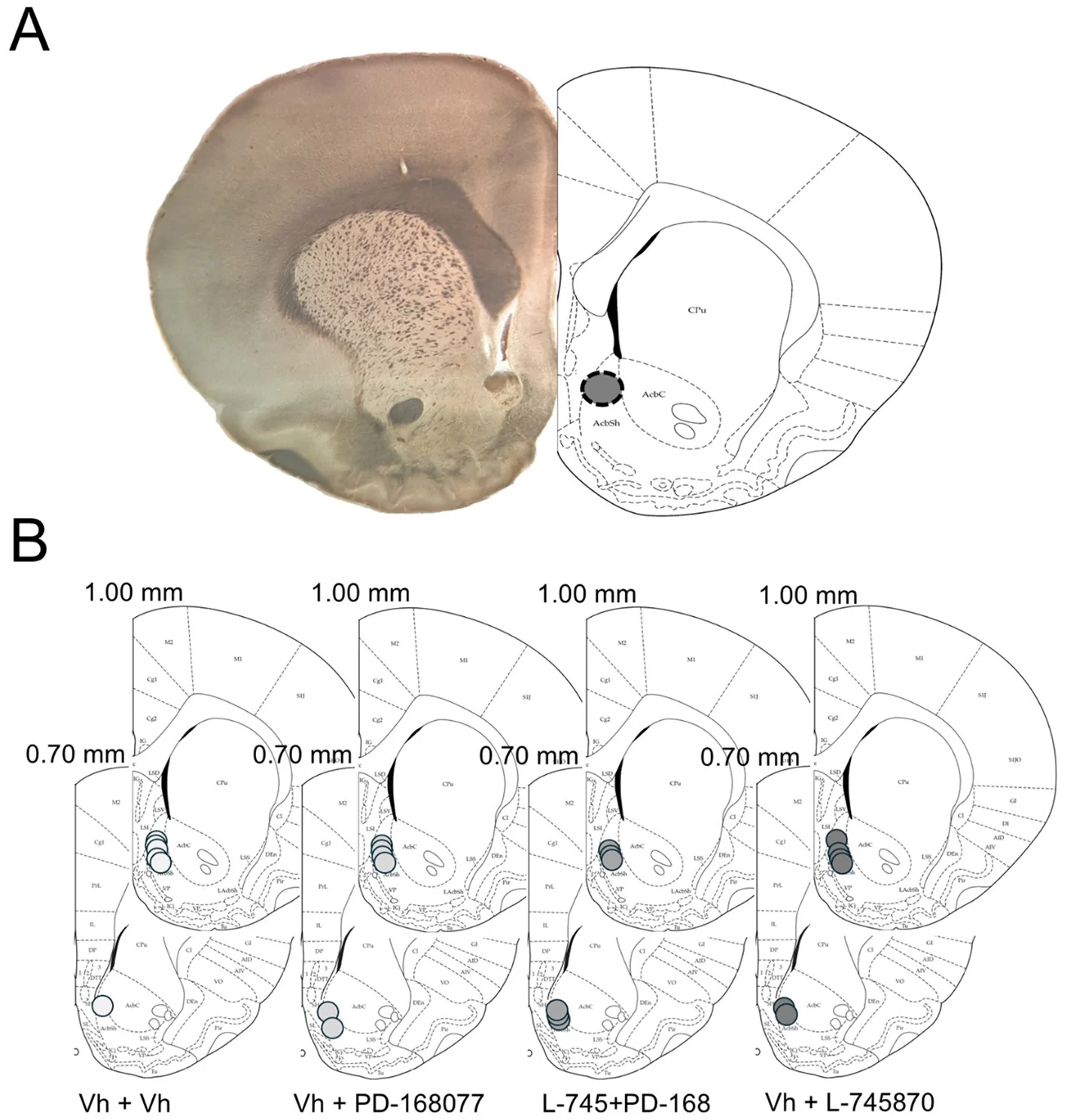
Schematic representation of intra-NAcSh drug injections. **(A)** The left panel shows a coronal section of the rat brain indicating the verified injection site within the NAcSh. The right panel represents a schematic illustration adapted from the Paxinos and Watson rat brain atlas, showing the injection site as a gray circle within the NAcSh. **(B)** Coronal brain sections were adapted from the stereotaxic atlas of Paxinos and Watson. The numbers displayed in the upper and middle portions of each section indicate the antero posterior distance relative to bregma (mm). The labels at the bottom of each panel correspond to the experimental groups.

After completing all behavioral procedures, the rats that underwent stereotaxic surgery were euthanized with an overdose of sodium pentobarbital. Then, their brains were dissected for histological verification. Coronal brain slices, 200 μm thick, were then prepared using a vibroslicer (Camden Instruments^®^, Leicestershire, UK) and examined under a stereomicroscope (Figure 2A). Only data from rats with injection sites located within the NAcSh, as verified using the rat brain atlas of Paxinos and Watson (1998), were included in the statistical analysis (Figure 2B). Animals with cannula misplaced outside the NAcSh were excluded from the analysis.

### 2.5 Statistical analysis

Data are expressed as the mean ± standard error of the mean (SEM). The assumptions of normality and homogeneity of variance were verified prior to statistical analysis. One-way analysis of variance (ANOVA) was used to analyze the effects of intra-NAcSh pharmacological treatments on food intake, active lever presses, reinforcers obtained, and breakpoint, followed by Tukey's *post hoc* test when appropriate. The effect of the systemic administration of the antagonist L-745,870 (i.p.) on operant training was analyzed using Student's *t*-test, whereas the effects of systemic administration of the D4R agonist PD-168,077 and the D4R antagonist L-745,870 were analyzed one-way ANOVA followed by Tukey's *post hoc* test. Statistical significance was set at *p* < 0.05. All analyses were performed using *GraphPad Prism* software version 8.0.2 (GraphPad Software, San Diego, CA, USA).

## 3 Results

### 3.1 Activation of D4Rs in the NAcSh increases motivation for palatable food

**Figure 3:**
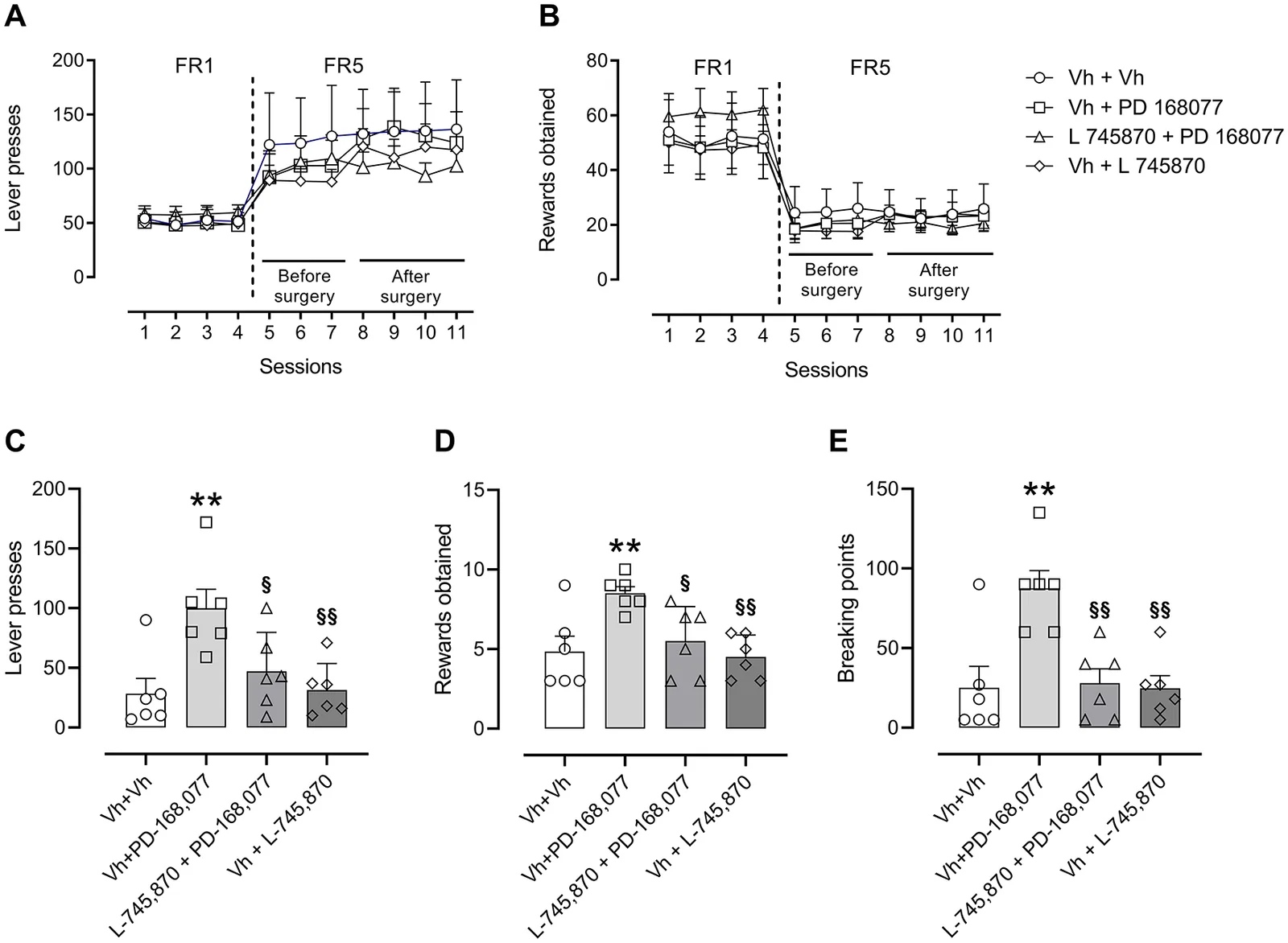
Intra-NAcSh activation of D4Rs enhances operant responding for palatable food, an effect prevented by L-745,870. lever presses **(A)** and rewards obtained across training sessions under fixed ratio 1 (RF1) and fixed ratio 5 (RF5) schedules of reinforcement. The dashed vertical line indicates the time of stereotaxic surgery for cannula implantation. Sessions 5–7 correspond to the pre-surgery period, whereas sessions 8–11 correspond to the post-surgery period. No significant differences were observed among the four experimental groups during any FR1 or FR5 training session. During the progressive ratio (PR) test, lever presses **(C)**, rewards obtained **(D)**, and breaking points **(E)** were evaluated. Bars represent the mean (± SEM). Animals received intra-NAcSh microinjections of vehicle (Vh + Vh, *n* = 7), PD-168,077 (Vh + PD-168,077, 0.1 μg, *n* = 7), PD-168,077 plus L-745,870 (0.1 μg each, *n* = 7), and L-745,870 (Vh + L-745,870, 0.1 μg, *n* = 7). Data were analyzed by one-way ANOVA followed by Tukey's *post hoc* test. **p<*0.01 (Vh*+*Vh* vs. *Vh*+*PD-168,077)*, ^**§**^*p* < 0.05 (Vh+PD-168,077 vs. L-745,870+PD-168,077, ^**§§**^*p* < 0.01 (Vh+PD-168,077 vs. Vh+L-745,870).

Before pharmacological testing, animals assigned to the four experimental groups underwent identical operant training under RF1 and RF5 schedules. Session-by-session analyses of lever presses and rewards obtained revealed no significant differences among groups before surgery or after recovery (Figures 3A, B), indicating comparable acquisition and maintenance of operant performance prior to drug administration.

To evaluate the role of D4Rs in the NAcSh in motivation for palatable food, the D4R agonist PD-168,077 was administered directly into this region before the PR test. Subsequently, the number of operant responses (lever presses), reinforcers earned, and the breakpoint were quantified. One-way ANOVA revealed a significant effect of treatment on the number of lever presses *F*(3,20) = 6.455; *p* = 0.0031], reinforcers obtained [*F*(3,20) = 5.941; *p* = 0.0046], and breakpoints [*F*(3,20) = 8.385; *p* = 0.008] (Figures 3C–E). *Post hoc* comparisons showed that intra-NAcSh administration of PD-168,077 significantly increased lever presses (*p* = 0.0048), rewards obtained (*p* = 0.0121), and breakpoint (*p* = 0.0026) compared with vehicle-treated animals. Importantly, pretreatment with the selective D4R antagonist L-745,870 completely prevented the stimulatory effects of PD-168,077 on all behavioral measures. In contrast, administration of L-745,870 *per se* did not significantly modify lever pressing, rewards obtained, or breakpoint relative to vehicle-treated animals.

### 3.2 Activation of D4Rs in the NAcSh increases standard chow feeding

**Figure 4:**
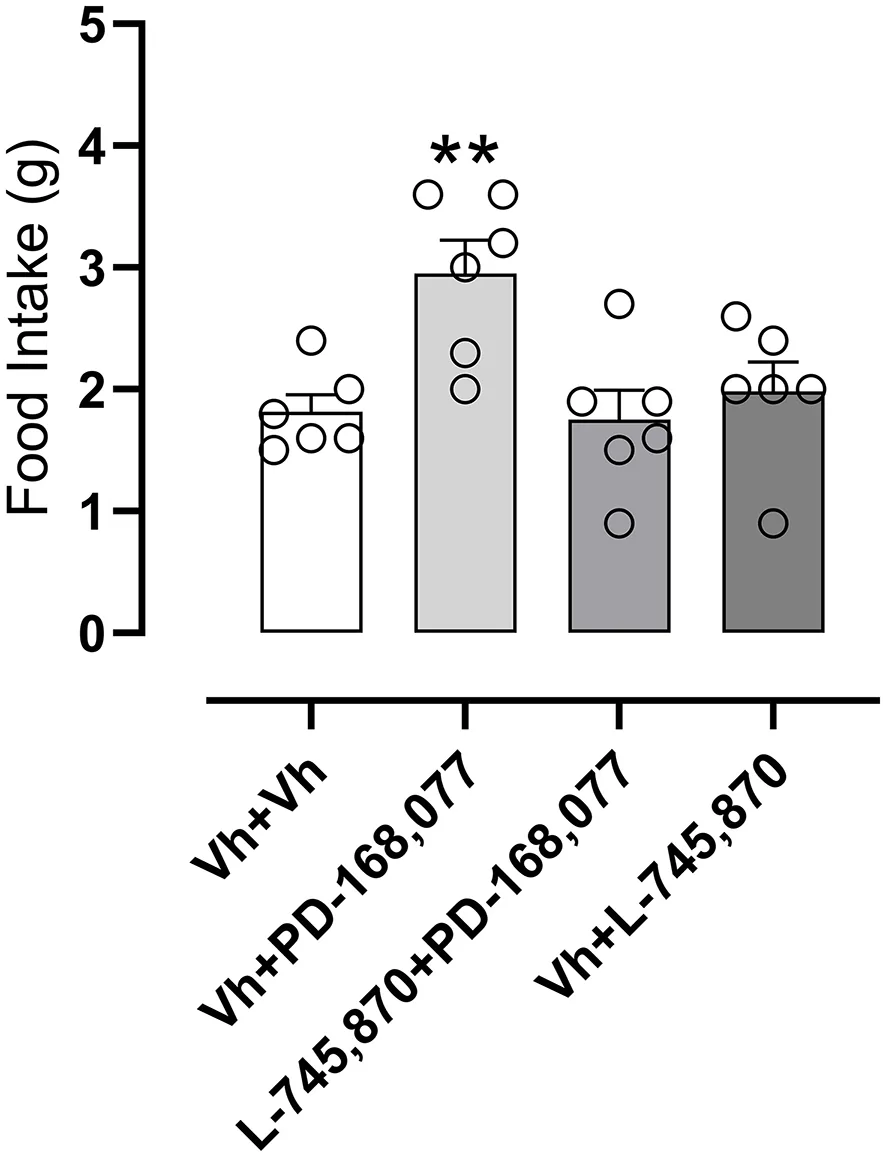
Activation of D4R in the NAcSh increases standard chow intake in satiated rats. This effect is completely reversed by the D4R antagonist L-745,870, which by itself does not affect food intake. Bars represent the mean (± SEM) consumption in grams. Vehicle (Vh + Vh, *n* = 6), PD-168,077 (Vh + PD-168,077, 0.1 μg, *n* = 6), PD-168,077 + L-745,870 (0.1 μg each, *n* = 6), L-745,870 (Vh + L-745,870, 0.1 μg, *n* = 6). When a significant ANOVA was obtained, Tukey's *post hoc* test was conducted. **p<*0.01*.

To determine the role of D4Rs in homeostatic feeding in the NAcSh, the D4R agonist PD-168,077 was administered into the NAcSh. Standard chow intake was then quantified during the first hour in satiated rats. The results are presented in Figure 4. Regarding standard chow intake, a one-way ANOVA revealed a significant treatment effect [*F*(3,20) = 5.958; *p* = 0.0045]. *Post hoc* analysis showed that the intra-NAcSh administration of the D4R agonist PD-168,077 significantly increased chow consumption (*p* = 0.0062) during pharmacological treatment, particularly at the beginning of the dark phase of the light-dark cycle in satiated rats with *ad libitum* access to food. Pretreatment with the D4R antagonist L-745,870 completely blocked the effect of PD-168,077; however, administration of the antagonist alone did not modify food intake.

### 3.3 Systemic administration of L-745,870 (0.1 mg/kg) reduces motivation for palatable food

**Figure 5:**
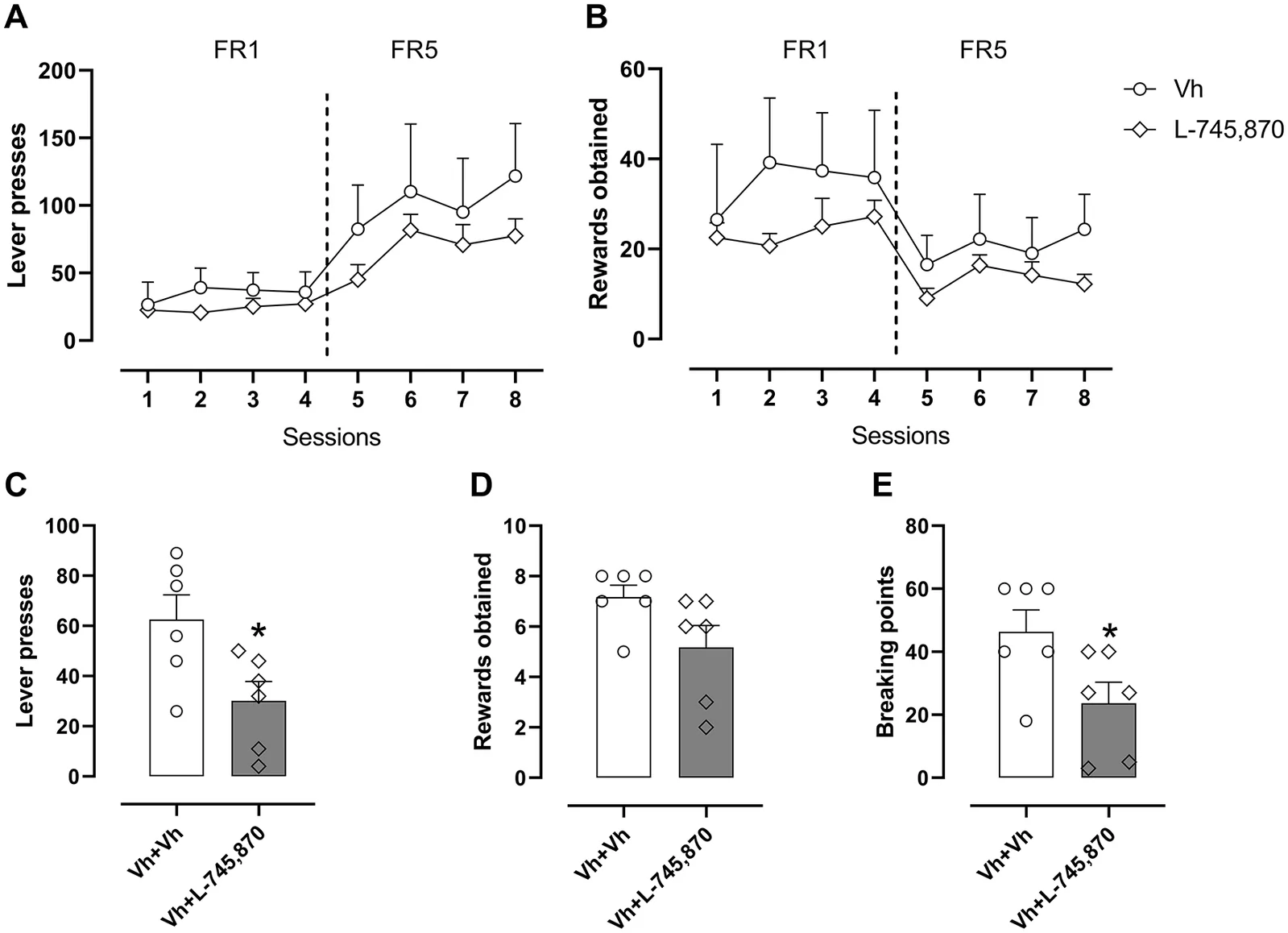
Systemic administration of the D4R antagonist L-745,870 reduces operant responding for palatable food. Lever presses **(A)** and rewards obtained **(B)** during training under RF1 and RF5 schedules. The dashed vertical line indicates the transition from RF1 to RF5. No significant differences were observed between the vehicle and L-745870 assigned groups during any training session. During the PR test, systemic administration of L-745,870 (0.1 mg/kg, i.p.) significantly reduced lever presses **(C)** and breakpoint **(E)** without significantly affecting the number of rewards obtained **(D)**. Bars represent the mean (± SEM) of the counts obtained. Vehicle (Vh, *n* = 6); L-745,870 (*n* = 6). Data were analyzed using Student's *t*-test. *p<*0.05*.

Prior to drug administration, animals were assigned to vehicle or L-745,870 treatment groups. Lever presses and rewards obtained were compared between the vehicle and L-745,870 groups at each RF1 and RF5 training session. No significant between-group differences were detected in any session (Figures 5A, B), confirming that both groups reached a comparable level of operant performance before drug administration.

To evaluate the effect of systemic D4R blockade on motivation to obtain palatable food, the selective D4R antagonist L-745,870 (0.1 mg/kg, i.p.) was administered before the progressive ratio test. Compared with vehicle-treated animals, systemic administration of L-745,870 significantly reduced the number of lever presses (*t* = 2.591, df = 10, *p* = 0.0269; Figure 5C) and significantly decreased the breakpoint (*t* = 2.357, df = 10, *p* = 0.0402; Figure 5E). In contrast, the number of reinforcers obtained was not significantly affected (*t* = 2.011, df = 10, *p* = 0.0720; Figure 5D), although a trend toward a reduction was observed in animals treated with L-745,870.

### 3.4 Systemic administration of L-745,870 (0.1 mg/kg) reduces standard chow food intake, whereas PD-168,077 (1 mg/kg) does not alter food intake

To determine the contribution of D4Rs in standard chow food intake, satiated rats received an intraperitoneal injection of either the D4R agonist PD-168,077 (1 mg/kg), the D4R antagonist L-745,870 (0.1 mg/kg), or vehicle. Food intake was measured during the first hour after treatment.

**Figure 6:**
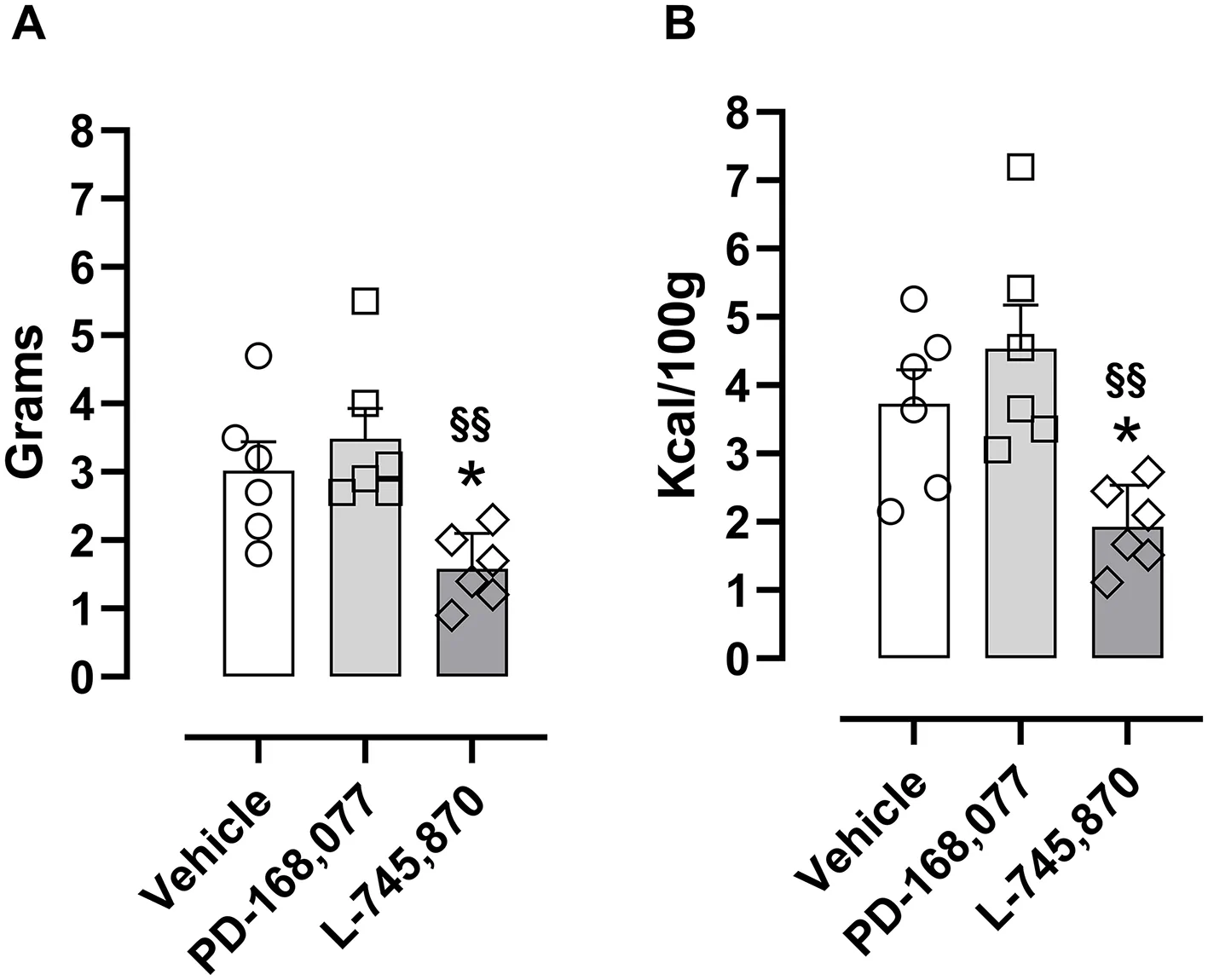
Effect of systemic D4R ligands on standard chow intake in satiated rats. Bars represent the mean (± SEM) of chow intake expressed as grams **(A)** and kcal/100 g body weight **(B)** during the first hour after treatment. Rats received vehicle (*n* = 6), PD-168,077 (1 mg/kg, *n* = 6), or L-745,870 (0.1 mg/kg, *n* = 6). Statistical analysis was performed using one-way ANOVA followed by Tukey's multiple comparisons test. *p<*0.05 (*Vehicle vs. L-745,870*)*, ^**§§**^*p* < 0.01 (PD-168,077 vs. L-745,870).

One-way ANOVA revealed a significant effect of treatment on chow intake [*F*(2,15) = 6.923; *p* = 0.0074]. *Post hoc* analysis showed that treatment with PD-168,077 did not significantly affect chow intake compared with the vehicle group. In contrast, L-745,870 significantly reduced chow intake compared with both the vehicle (*p* < 0.05) and PD-168,077 (*p* < 0.01) groups (Figure 6A).

Because caloric intake reflects the energetic value of the food consumed, intake was also expressed as kcal/100 g body weight (Figure 6B). The pattern of results was consistent that observed for food intake expressed in grams. PD-168,077 did not differ from the vehicle group, whereas L-745,870 significantly reduced caloric intake compared with both the vehicle and PD-168,077-treated groups.

## 4 Discussion

In the present study, we found that administering the D4R agonist PD-168,077 into the NAcSh significantly increased motivation for palatable food. This was evidenced by an increase in lever presses, number of reinforcers obtained, and breakpoint values. This effect was reversed by the D4R antagonist L-745,870. Additionally, systemic blockade of D4Rs with L-745,870 (0.1 mg/kg) reduced motivation for palatable food. To our knowledge, this is the first study to report the effects of systemic D4R antagonism on operant behavior associated with palatable food seeking. Furthermore, intra-NAcSh activation of D4Rs with PD-168,077 increased chow intake; this effect was also reversed by the D4R antagonist L-745,870. However, systemic D4R activation with PD-168,077 (1 mg/kg) did not significantly reduce chow intake during the first hour of recording, whereas systemic blockade with L-745,870 (0.1 mg/kg) decreased chow intake.

Traditionally, food intake regulation was believed to occur primarily in the hypothalamus, which is the main regulator of feeding based on homeostatic needs. However, more recent research has focused on other brain regions, particularly those involved in the hedonic aspects of feeding. One such region is the NAc, which acts as a neural hub at the interface between homeostatic and hedonic feeding circuits (Marinescu and Labouesse, 2024). Anatomical interconnections between homeostatic and hedonic neurocircuits suggest both systems contribute to a complex motivational framework, indicating they are not functionally dissociated (Rossi and Stuber, 2018).

Notably, D4Rs in the NAcSh appear to participate in both reward-related processes. We previously demonstrated that activation of D4Rs in the NAcSh of satiated rats with *ad libitum* access to palatable food increased hypercaloric food intake, consistent with an enhancement of the hedonic “liking” component of feeding behavior (Cruz-Trujillo et al., 2024). The present findings extend these observations by showing that D4R activation in the NAcSh also increases motivation to obtain sucrose pellets under an operant paradigm, supporting a role for D4Rs in the incentive salience or motivational “wanting” component of reward.

### 4.1 Administering PD-168,077 into the NAcSh increases lever pressing, reinforcers, and breakpoints

Motivation to consume sucrose pellets can induce sufficient dopamine release within the NAcSh (Cone et al., 2014). Previous studies have shown that intra-NAcSh amphetamine infusion increases lever pressing and breakpoints in non-food-deprived animals under operant schedules (Zhang et al., 2003). This dopaminergic increase may diffuse beyond the immediate synaptic site and activate presynaptic D4Rs on cortical afferent terminals (Svingos et al., 2000). D4Rs are coupled to inhibitory G-proteins and suppress cAMP formation by inhibiting adenylyl cyclase. D4Rs reduce L-type voltage-gated calcium currents, and the Gβγ subunit interacts with inwardly rectifying potassium channels. Together, these mechanisms facilitate inhibitory signaling (Oak et al., 2000; Rondou et al., 2010), which suggests that D4R activation may reduce glutamate release onto NAcSh neurons (Bonaventura et al., 2017; González et al., 2012).

The NAcSh receives dense glutamatergic afferents from the medial prefrontal cortex (mPFC), basolateral amygdala (BLA), and ventral hippocampus (vH), which converge together with dopaminergic projections from the VTA onto the dendrites of MSNs. This anatomical organization provides an ideal substrate for dopamine to directly modulate glutamatergic transmission within the NAcSh, particularly in D1-expressing MSNs (Yu et al., 2022). These glutamatergic inputs contribute to decision-making, emotionally salient information, and memory-related processes (Xu et al., 2024), while also playing a critical role in the regulation of consummatory feeding behavior (Prado et al., 2016). Importantly, presynaptic D4Rs have been identified on corticostriatal glutamatergic terminals, where their activation reduces glutamate release onto NAc neurons (Bonaventura et al., 2017; González et al., 2012). Similar D2-like receptor-mediated modulation has also been described for glutamatergic afferents arising from the vH (Yan and Mogenson, 1986; Hunt et al., 2005). Together, these findings suggest that D4Rs positioned on glutamatergic terminals are ideally situated to regulate excitatory drive onto MSNs and, consequently, influence downstream circuits involved in both motivational and consummatory aspects of feeding.

**Figure 7:**
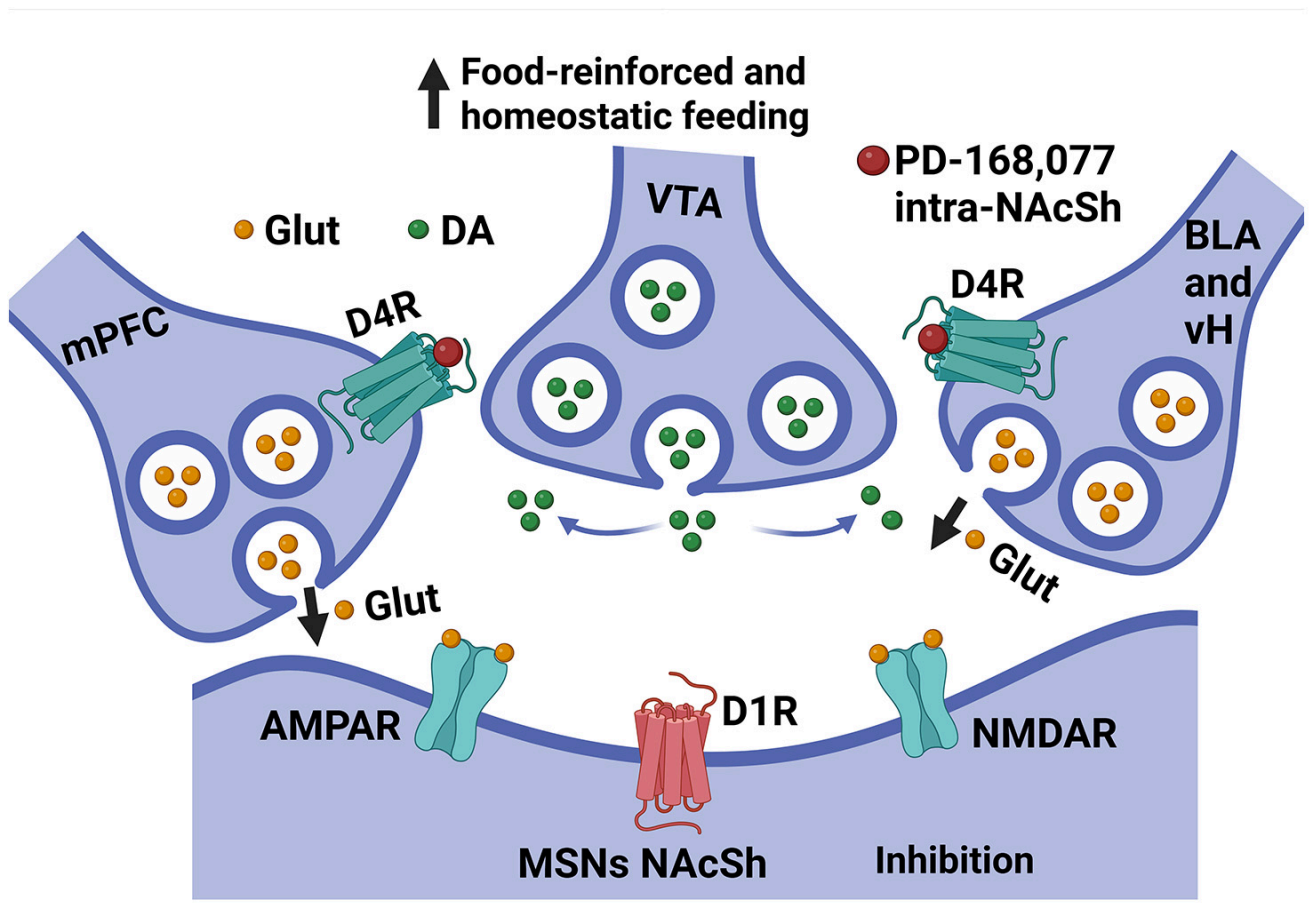
Proposed mechanism by which intra-NAcSh D4R activation modulates food-reinforced and homeostatic feeding. Activation of dopamine D4 receptors (D4Rs) by PD-168,077 is proposed to reduce glutamatergic input from cortical and limbic afferents, including the medial prefrontal cortex (mPFC), basolateral amygdala (BLA), and ventral hippocampus (vH). This reduction in glutamatergic transmission decreases the firing rate of D1-expressing GABAergic MSN (MSND1^+^) in the nucleus accumbens shell (NAcSh) by limiting AMPA receptors (AMPAR) and NMDA receptors (NMDAR) mediated excitation. The resulting inhibition of MSNs leads to disinhibition of downstream hypothalamic circuits, including the lateral hypothalamus (LH), and ventral pallidum thereby promoting a robust homeostatic feeding response and motivation for palatable food, respectively. Dopaminergic input from the ventral tegmental area (VTA) is also shown, highlighting the interaction between dopaminergic and glutamatergic signaling in the regulation of food-reinforced and homeostatic feeding. Created in https://BioRender.com.

Based on this framework, we propose that intra-NAcSh PD-168,077 activated presynaptic D4Rs located on glutamatergic terminals arising from the BLA, vH, and mPFC that converge onto NAcSh MSNs. Activation of these Gi/o-coupled receptors would reduce glutamate release, thereby decreasing the excitatory drive onto MSNs. Consequently, reduce MSNs activity would decrease the inhibitory output from the NAcSh to the ventral pallidum (VP), a major downstream target critically involved in incentive motivation and reward-related behaviors. Evidence indicates that attenuation of inhibitory transmission from the NAc indirect pathway MSNs to the VP is sufficient to increase willingness to work for food rewards, resulting in higher lever pressing and breakpoints values under PR schedules (Gallo et al., 2018). Thus, disinhibition of VP neurons may account for the enhanced motivational performance observed after intra-NAcSh PD-168,077 administration. The VP is also recognized as a key integrative node within the mesolimbic circuitry, processing motivational and reward-related information that contributes to food-directed behaviors (Castro et al., 2015). Therefore, D4R-mediated modulation of the NAcSh-VP pathway provides a plausible neural substrate through which local D4R activation increases in lever pressing, reinforcers earned, and breakpoint following PD-168,077 administration (Figure 7). The pretreatment with the selective D4R antagonist L-745,870 10 min before PD-168,077 prevent receptor activation and abolished these behavioral effects, supporting the conclusion that they specifically mediated by local D4Rs within the NAcSh rather than non-specific drug actions.

### 4.2 Administering PD-168,077 directly into the NAcSh significantly increases standard chow intake

Treatment with the D4R agonist PD-168,077 significantly increases standard chow intake when administered directly into the NAcSh. This effect was reversed by pretreating with the selective D4R antagonist L-745,870. However, administering the antagonist alone had no significant effect on food intake in satiated rats.

Dopamine is known to modulate glutamate release within NAc neurons. Electrophysiological recordings from medial NAc output neurons during corticolimbic afferent stimulation reveal excitatory responses that are attenuated by dopamine application (Pennartz et al., 1992). Furthermore, anatomical studies indicate that dopaminergic terminals projecting to the NAc do not form axoaxonic contacts with excitatory afferents. Instead, they primarily synapse onto distal dendritic spines of MSNs (Smith and Bolam, 1990). These same dendritic spines also receive excitatory inputs from cortical regions, such as the mPFC (Sesack and Pickel, 1990).

In addition, immunocytochemical studies have demonstrated the presence of D4Rs in pyramidal projection neurons of the frontal cortex (Mrzljak et al., 1996), which suggests that these receptors may be transported to presynaptic terminals within the NAcSh (Svingos et al., 2000). Food intake increases dopamine release from the VTA to the NAcSh. This increase may activate presynaptic D4Rs on cortical afferent terminals, reducing glutamate release onto NAcSh neurons (Bonaventura et al., 2017; González et al., 2012).

Activation of D4Rs by PD-168,077 would be expected to reduce the firing rate of NAcSh neurons by decreasing glutamatergic input in this region. This mechanism may explain the observed increase in standard chow intake. Blocking AMPA and kainate receptors in the NAcSh reduces MSN excitation (Figure 7). This leads to the disinhibition of the lateral hypothalamus (LH), which triggers a robust homeostatic feeding response (Kelley and Swanson, 1997; Maldonado-Irizarry et al., 1995).

### 4.3 Systemic administration of L-745,870 reduces lever pressing, breakpoint, and chow intake

Unlike intra-NAcSh administration, systemic D4R blockade simultaneously targets D4Rs expressed across multiple interconnected nodes of the mesocorticolimbic network. Although previous studies reported that systemic administration of the selective D4R antagonist L-745,870 (0.1–3 mg/kg, i.p.) did not alter food-seeking behavior in an operant paradigm (Yan et al., 2012), and that D4R knockout mice do not differ from wild-type animals in food-reinforced operant responding (Thanos et al., 2010), the contribution of D4Rs to the motivational aspects of feeding has remained unclear. The present findings provide the first evidence that systemic administration of L-745,870 significantly reduces motivation for palatable food, as reflected by decreased lever pressing and breakpoints. These observations extend our previous work demonstrating that direct intra-NAcSh administration of L-745,870 similarly reduced operant responding for palatable food (Cruz-Trujillo et al., 2020), suggesting that D4Rs regulate incentive motivation through both local and distributed neural mechanisms.

Evidence accumulated over the last decade indicates that D4Rs exert inhibitory control over glutamatergic signaling within the NAcSh. Systemic administration of L-745,870 (1 mg/kg, i.p.) abolishes the differential effects of methylphenidate on dopamine release in the NAcSh of wild-type and D4.7 mice (Bonaventura et al., 2017), while D4R knockout mice exhibit reduced dopamine release accompanied by increased extracellular glutamate levels (Thomas et al., 2007, 2008). Together, these findings support the view that D4Rs normally restrain excitatory drive within accumbal circuits.

D4Rs are abundantly expressed in the mPFC, BLA, vH, and nucleus accumbens, where they regulate glutamatergic transmission and synaptic plasticity (Defagot et al., 1997; Khan et al., 1998; Ohshiro et al., 2011; Svingos et al., 2000). In the mPFC, presynaptic D4Rs located on corticostriatal terminals reduce glutamate release onto NAc neurons (Bonaventura et al., 2017; González et al., 2012). Likewise, D4Rs within the BLA regulate excitatory and inhibitory transmission involved in emotionally salient associative processing (Floresco and Tse, 2007; Laviolette et al., 2005). Supporting the functional relevance of this pathway, blockade of D2-like receptors with haloperidol attenuates excitatory postsynaptic potential recorded in NAc neurons following BLA stimulation (Yim and Mogenson, 1986), suggesting that dopamine receptors of the D2-like family, including D4Rs, modulate amygdalo-accumbal glutamatergic transmission. In the vH, D4Rs contribute to synaptic plasticity and gamma network oscillations that influence contextual processing (Andersson et al., 2012; Navakkode et al., 2017). Therefore, systemic administration of L-745,870 is likely to increase excitatory drive across several glutamatergic afferents that converge onto NAcSh MSNs.

Our previous findings support this interpretation. Intra-NAcSh administration of the D4R agonist PD-168,077 increased palatable food intake in satiated rats, whereas activation of NMDA receptors prevented this effect (Cruz-Trujillo et al., 2024). Conversely, treatment with the AMPA/kainate receptor antagonist CNQX produced effects like those of the D4R agonist on palatable food consumption. AMPA receptors in the NAc play a critical role in hedonic feeding because they colocalize with D1 receptors in MSNs and modulate AMPA receptor trafficking (Italia et al., 2025). Microinjection of AMPA into the NAcSh reduces palatable food intake in pre-satiated rats, an effect countered by D4R activation (Cruz-Trujillo et al., 2024). Furthermore, activating AMPA receptors in the NAcSh decreases breakpoints and lever pressing in rats subjected to short-term food deprivation (Mena et al., 2013).

The convergence of these glutamatergic afferents onto NAcSh MSNs provides an anatomical substrate through which systemic D4R blockade may simultaneously influence multiple downstream circuits. Increased excitatory drive is likely to activate D1-MSNs, thereby strengthening inhibitory GABAergic output from the NAcSh toward both the VP, a structure critically involved in effort-related motivation (Gallo et al., 2018), and lateral hypothalamic (LH) GABAergic neurons that regulate consummatory feeding behavior (O'Connor et al., 2015; Prado et al., 2016). Consequently, systemic D4R antagonism may reduce both the motivational value of food and the execution of feeding behavior by acting on partially overlapping, rather than completely independent neural circuits. This interpretation is consistent with evidence showing that inhibition D1-MSN transmission reduces lever-pressing force during food-seeking behavior and mitigates high-fat diet-induced weight gain (Matikainen-Ankney et al., 2023).

Importantly, the reduction in operant responding and chow intake is unlikely to reflect nonspecific motor impairment, as systemic administration of L-745,870 does not alter locomotor activity (Patel et al., 1997; Erlij et al., 2012). Instead, the present findings indicate that D4R blockade selectively attenuates both the motivational drive to obtain palatable food and the consumption of standard chow, supporting a role for D4Rs in regulating multiple components of feeding behavior.

Future studies should evaluate whether systemic treatment with the D4R antagonist also decreases palatable food intake under satiated rats. Recent evidence indicates that intracerebroventricular administration of L-745,870 decreases sucrose consumption in a dose-dependent manner, and alters the microstructure of licking behavior, suggesting that D4Rs also contribute to hedonic processing (López-Alonso et al., 2023). Likewise, systemic L-745,870 reduces binge-like consumption of palatable food in mice, an effect enhanced by CB2 receptor activation (Rodríguez-Serrano et al., 2025). The interaction between D4Rs and CB2 receptors may represent an important mechanism that modulates reward-driven feeding behavior. Emerging evidence further suggests that functional interactions between D4Rs and μ-opioid receptors within mesolimbic circuits play an important role in reward-based motor learning and habit acquisition associated with addiction (Guidolin et al., 2023). Together, these findings raise the possibility that D4Rs regulate multiple components of reward-driven feeding, extending beyond motivational processes alone.

## 5 Conclusion

Together, these findings suggest that motivational and consummatory aspects of feeding rely on overlapping D4R-depedent mesolimbic circuitry. Both behavioral components appear to share common upstream mechanisms involving D4R-mediated modulation of glutamatergic inputs from the medial prefrontal cortex, basolateral amygdala, and ventral hippocampus to the NAcSh. However, these signals are likely conveyed through partially divergent downstream pathways, with the ventral pallidum primarily mediating incentive motivation (“wanting”) and hypothalamic circuits contributing to the regulation of consummatory feeding. Consistent with this framework, local activation of D4Rs within the NAcSh by PD-168,077 enhanced both operant responding for palatable food and homeostatic food intake, whereas systemic D4R blockade with L-745,870 reduced both motivational responding and standard chow consumption. Collectively, these findings identify D4Rs as important modulators of mesolimbic glutamatergic signaling that coordinate reward-related associative processes and feeding behavior through interconnected, yet partially specialized, neural circuits.

## Data Availability

The raw data supporting the conclusions of this article will be made available by the authors, without undue reservation.
